# Time-Series-Based Co-Expression Network Analysis Reveals Key Regulatory Modules and Hub Genes in Salt-Tolerant Wheat Under Salt Stress

**DOI:** 10.3390/cimb48030317

**Published:** 2026-03-16

**Authors:** Guiqiang Fan, Jianan Huang, Hong-Jin Wang, Yuxiang Huo, Peiyu Liu, Uzair Ullah, Guohang Hu, Munib Ahmad, Abdullah Shalmani, Hui Fang, Tianrong Huang

**Affiliations:** 1 Crop Research Institute of Xinjiang Uygur Autonomous Region Academy of Agricultural Sciences, Urumqi 830002, China; 2 College of Life Sciences, Northwest A&F University, Yangling 712100, China

**Keywords:** wheat, salt stress, time-series transcriptome, WGCNA, co-expression networks, hub genes

## Abstract

Salt stress severely constrains wheat growth and yield by inducing osmotic imbalance, ion toxicity, and excessive accumulation of reactive oxygen species (ROS). Although salt-tolerant cultivars can adapt through rapid signaling transduction and maintenance of cellular homeostasis, the underlying dynamic regulatory networks remain insufficiently characterized. In this study, we reanalyzed publicly available time-series RNA-seq data (0, 1, 3, 6, 12, and 24 h) from the salt-tolerant wheat cultivar Xiaoyan22 under salt stress and constructed a time-series-based co-expression network using weighted gene co-expression network analysis (WGCNA). Multiple gene modules were identified, among which the black module showed significant positive correlations with both salt treatment (treatment_bin) and stress duration (time_h). This module displayed a progressively increasing eigengene expression pattern throughout the stress period. Gene significance (GS) was positively correlated with module membership (MM), facilitating the identification of highly connected hub genes within this module. Functional enrichment analysis indicated that genes in the black module were primarily associated with DNA replication and genome stability maintenance, RNA metabolic regulation, phenylpropanoid metabolism, and cuticle/suberin/wax biosynthesis. Physiological analysis further revealed enhanced activities of superoxide (SOD), peroxide (POD), and catalase (CAT), enhanced accumulation of proline and soluble sugars, and a time-dependent increase in MDA under salt stress. qRT-PCR confirmed significant induction of candidate genes, including a ZAR1-like receptor kinase, Remorin, and NETWORKED 1D. Collectively, these findings integrate co-expression network inference with physiological and molecular validation, providing candidate regulators and pathways for understanding salt tolerance and supporting future molecular breeding efforts.

## 1. Introduction

Salinity stress, a major abiotic stress, is one of the greatest limiting factors for crop growth and global agricultural production, particularly in arid and semi-arid regions where soil salinization is intensifying [1,2]. Wheat (*Triticum aestivum* L.), one of the world’s most important staple crops, is highly vulnerable to salinity stress; therefore, maintaining stable wheat yields is crucial for food security [3,4]. Under saline conditions, wheat typically exhibits inhibited vegetative growth, premature leaf senescence, impaired root development, and reduced grain filling and maturation, ultimately leading to substantial losses in yield and grain quality [5,6]. Physiologically, salinity stress primarily damages plants through osmotic stress caused by high Na^+^/Cl^−^, ion toxicity, and disruption of K^+^/Na^+^ homeostasis due to excessive Na^+^/Cl^−^ accumulation, and oxidative injury resulting from stress-induced overproduction of reactive oxygen species (ROS), which promotes membrane lipid peroxidation and membrane damage [7,8]. Accordingly, elucidating the molecular regulatory mechanisms that underlie salt tolerance is essential for improving wheat performance on saline soils and for facilitating the genetic development of salt-tolerant varieties.

The plant response to salinity stress is highly dynamic and stage-dependent. In the early phase, plants rapidly perceive salt stimuli through membrane receptors, ion channels, and Ca^2+^ signaling, which activate MAPK cascades and hormone signaling pathways such as ABA and trigger large-scale transcriptional reprogramming via diverse transcription factors [9,10]. In later phases, plants establish cellular homeostasis and long-term adaptation through ion homeostasis regulation (e.g., Na^+^ efflux and vacuolar sequestration), accumulation of osmoprotectants (proline and soluble sugars), reinforcement of antioxidant enzyme systems (SOD, POD, CAT), and cell wall/cuticle remodeling [11,12,13,14,15]. Because salt stress responses are temporally structured, differential expression analyses at a single time point often fail to capture coordinated regulation across stages or identify key drivers of long-term adaptation [16]. Therefore, integrating time-series transcriptomics with network-level approaches to identify core modules and hub genes is an important direction for dissecting salt stress-associated regulatory response and facilitating breeding applications [17,18].

Weighted gene co-expression network analysis (WGCNA) provides a systems biology framework to cluster genes into co-expression modules based on expression similarity and to establish “module–trait” relationships, offering advantages over traditional differential expression analyses that focus primarily on individual genes [19]. In addition, WGCNA enables the identification of highly connected hub genes within key modules, which often represent critical regulators or core functional genes. WGCNA has been widely applied in plant stress research to uncover regulatory frameworks underlying complex responses [20,21]. However, systematic characterization of time-series-based co-expression networks and key modules in salt-tolerant wheat under time-series salinity stress remains limited, particularly studies that integrate network inference with physiological measurements and molecular validation [22,23].

Based on this, our study uses RNA-seq data from a time-series salt stress experiment on the salt-tolerant wheat variety Xiaoyan22 to construct a time-series-based co-expression network under salt stress using WGCNA. The aims are to: (1) identify key co-expression modules significantly associated with salt treatment and stress progression; (2) screen for hub genes within key modules and analyze their dynamic expression characteristics; (3) reveal the potential biological functions of key modules through GO and pathway enrichment analysis; and (4) provide experimental support for network inference results by combining measurements of antioxidant and osmotic regulation-related physiological indicators and qRT-PCR validation of candidate genes. Through this research, this study will reveal the systematic regulatory framework of salt stress response in salt-tolerant wheat from a co-expression network perspective, and provide candidate gene resources and theoretical clues for subsequent functional analysis and molecular breeding.

Although the original dataset has been analyzed using differential expression approaches, coordinated transcriptional programs and module-level regulatory dynamics across stress progression remain insufficiently characterized. Time-series-based co-expression network analysis enables the identification of core modules and highly connected hub genes associated with stress duration and treatment intensity. Therefore, reanalyzing this dataset from a network perspective may provide additional systems-level insights into salt stress-associated transcriptional organization in a salt-tolerant wheat genotype.

## 2. Materials and Methods

### 2.1. RNA-Seq Data Source and Preprocessing

This study reanalyzed publicly available time-series RNA-seq data of the salt-tolerant wheat variety Xiaoyan22 under salt stress. The dataset was originally generated and published by [24], where the primary focus was differential gene expression analysis across salt treatment time points. The raw sequencing data were obtained from the China National Center for Bioinformation (CNCB) database under project number PRJCA033247 (DOI: 10.1016/j.ygeno.2025.111114). According to the original publication [24], total RNA was extracted using the Qiagen RNeasy Plant Mini Kit (QIAGEN N.V., Venlo, The Netherlands), and sequencing libraries were constructed and sequenced on the Illumina HiSeq X Ten platform with 150 bp paired-end reads. Sequencing services were provided by Novogene (Beijing, China). Raw sequencing reads were subjected to quality control using fastp v0.23.2 to remove adaptor sequences and low-quality reads. Clean reads were aligned to the wheat reference genome using HISAT2 v2.2.0, and transcript assembly and expression quantification were performed using StringTie v2.2.1.4 Gene expression levels were calculated as FPKM values. The FPKM expression matrix was used as input for WGCNA analysis. Because FPKM values account for sequencing depth and gene length normalization, no additional global normalization was applied. Genes with zero expression across all samples were removed before network construction. In the present study, we applied a time-series-based weighted gene co-expression network analysis (WGCNA) framework to systematically investigate module-level regulatory patterns and module–trait associations, thereby providing complementary insights beyond traditional differential expression analysis. The dataset consisted of salt-treated (Salt) and control (CK) samples collected at 0, 1, 3, 6, 12, and 24 h, with three biological replicates per treatment at each time point. After preprocessing and expression quantification, the FPKM expression matrix was used for downstream co-expression network analysis. Also constructed was a new file with traits representing each sample. In the trait file, the treatment variable was converted to a binary treatment, designated as treatment_bin (CK = 0, Salt = 1), while the time variable was designated time_h, in hours, for future analysis of trait-module associations.

### 2.2. Physiological Indicator Measurement

Seedlings of Xiaoyan22 were grown in vermiculite mixture (3:1, *v*/*v*) under controlled greenhouse conditions (25 ± 2 °C day/20 ± 2 °C (night), 16 h light/8 h dark photoperiod, relative humidity 60–70%). Seeds were surface sterilized prior to sowing and irrigated with half-strength Hoagland nutrient solution every three days. At the three-leaf stage, plants were subjected to salt stress by irrigation with 150 mM NaCl solution for 24 h. During the treatment period, no additional fertilization was applied. Control plants were irrigated with distilled water. Samples were collected at 0 h, 6 h, and 24 h after salt treatment, with three biological replicates per treatment. Enzyme activities (SOD, POD, CAT) and metabolite contents (proline, soluble sugars, MDA) were measured using commercial assay kits (Solarbio, Beijing, China) following the manufacturer’s instructions.

### 2.3. qRT-PCR Verification

To examine the effects of salt stress on candidate gene expression, the genes *TraesCS7A02G245400*, *TraesCS4B02G076500,* and *TraesCS4B02G276800* were selected for qRT-PCR analysis. For this analysis, samples were collected from three time points on both control and salt-treated plants (0 h, 6 h, 24 h), with three biological replicates at each time point (*n* = 3). Total RNA was extracted using a commercial RNA extraction kit (RNA Easy Fast Plant Tissue Kit No. DP452, manufactured by TIANGEN BIOTECH (BEIJING) Co., Ltd., Beijing, China), and RNA concentration and purity were determined by measuring the A260/280 ratio using a NanoDrop spectrophotometer (Thermo Fisher Scientific, Wilmington, DE, USA). First-strand cDNA was synthesized using a reverse transcription kit (HiScript IV All-In-One Ultra RT SuperMix for qPCR kit No. R433-01, Vazyme, Nanjing, China) according to the manufacturer’s instructions. The qRT-PCR was carried out using the previous study [25], where reactions were performed with a SYBR Green fluorescent quantitative system by using the CFX96 Real-Time PCR Detection System (Bio-Rad, Hercules, CA, USA) and included three technical replicates for each sample. Relative levels of gene expression were calculated using the 2^−ΔΔCt^ method, with *TaActin* used as the internal reference gene. Primer sequences for target genes and *TaActin* are provided in Supplementary Table S1.

### 2.4. Bioinformatics Analysis (WGCNA Network Construction and Functional Analysis)

The first step in the evaluation of the expression profiles of the samples was to evaluate the consistency of the samples using graphical methods, including box plots of the expression levels across the samples, PCA, hierarchical clustering, and a correlation heatmap. In this analysis, we calculated the weighted gene co-expression network (WGCNA, R version 1.72-1). To determine the value of “soft threshold” (power) that produced scale-free topology using WGCNA’s method (the “pickSoftThreshold” function), we generated the adjacency matrix from the data and calculated the TOM from that. The TOM-based distance matrix was used for hierarchical clustering and to determine the presence of co-expression modules using the “cutting dynamic tree” method (i.e., minModuleSize = 30) [26]. To identify the final co-expression modules, we merged those with similar ME (cutHeight = 0.25).

To screen for key modules related to salt stress, the Pearson correlation coefficient between each module’s ME and sample trait variables (treatment_bin, time_h) was calculated. *p*-values were adjusted using the Benjamini–Hochberg method to control the false discovery rate, and modules with adjusted *p*-values < 0.05 were considered significantly correlated modules. Within the key modules, module membership (MM/kME) and gene significance (GS) were further calculated, and high-connectivity hub genes (Top20/Top100) were selected based on kME values. The top20 genes were used for visualization of expression patterns, while the top100 genes were used for extended functional annotation and network exploration. To further refine candidate selection within the large black module, genes with high module membership (MM > 0.8) and relatively high gene significance (GS > 0.4) were prioritized for focused interpretation, highlighting genes most strongly associated with both module connectivity and salt stress traits.

GO and pathway enrichment analysis of key module genes was performed using clusterProfiler (v4.8.0) and enrichplot (v1.20.0) [27,28]. Multiple testing correction was performed using the Benjamini–Hochberg method, and the significance threshold was set to FDR < 0.05 [29]. Module network data was exported in Cytoscape format and visualized in Cytoscape (v3.9.1) [30]. All statistical analyses and plotting were performed in the R (v4.3.1) environment, mainly using the packages ggplot2 (v3.4.4) and pheatmap (v1.0.12) [31,32].

## 3. Results

### 3.1. RNA-Seq Data Quality Assessment and Sample Consistency Testing

**Figure 1:**
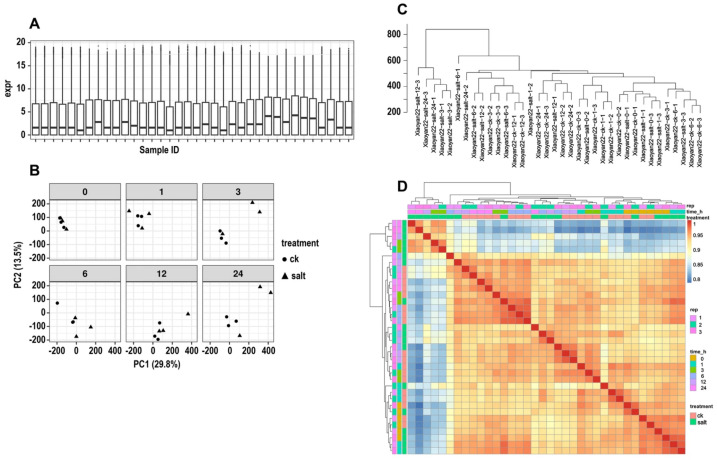
RNA-seq data quality assessment and sample consistency testing. (**A**) Boxplot of normalized expression levels. Distribution of normalized gene expression levels for each sample in the Xiaoyan22 salt stress time series (0, 1, 3, 6, 12, 24 h). (**B**) Principal component analysis (PCA) to assess overall sample differences and reproducibility. PCA analysis was performed on all samples based on the normalized expression matrix. (**C**) Sample clustering dendrogram. Hierarchical clustering analysis was performed on all samples, and a dendrogram was generated. (**D**) Inter-sample correlation heatmap. Pearson correlation coefficients between samples were calculated, and a heatmap was generated.

To ensure the reliability of subsequent co-expression network analysis, this study first performed a quality assessment of the transcriptome expression matrix of Xiaoyan22 under salt stress time series (0, 1, 3, 6, 12, 24 h). The expression distribution box plot showed that the normalized expression levels of each sample were generally consistent, with no obvious distribution shifts or abnormal samples, indicating good consistency in sequencing depth and normalization (Figure 1A). Principal component analysis (PCA) results further showed that PC1 and PC2 explained 29.8% and 13.5% of the total variance, respectively. Samples from different treatments/time points exhibited a separation along PC1 primarily reflecting treatment differences, while biological replicates clustered tightly, suggesting that salt stress and time course were major contributors to transcriptome pattern and had high reproducibility (Figure 1B). Hierarchical clustering analysis also did not reveal any outlier samples, and each replicate sample clustered preferentially with the same treatment or similar time points (Figure 1C). In addition, the sample correlation heatmap showed a high Pearson correlation coefficient among biological replicates (r > 0.95), indicating strong intra-group consistency (Figure 1D). In summary, the above results collectively indicate that the time-series RNA-seq data are of stable quality and good reproducibility, and can be used to construct a highly reliable gene co-expression network and conduct subsequent analyses.

### 3.2. WGCNA Construction of Co-Expression Network and Optimal Soft Threshold Selection

**Figure 2:**
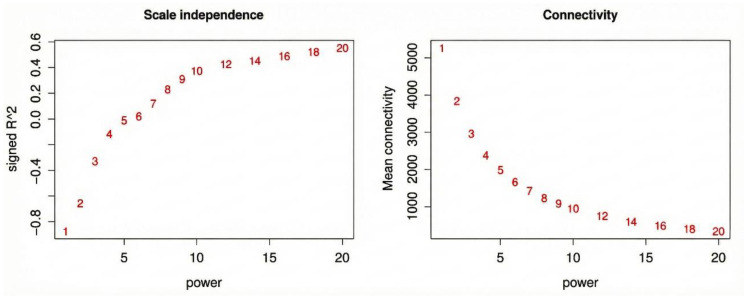
WGCNA soft-threshold power selection. The scale-free topology fit index and mean connectivity were evaluated under different soft-threshold power values. A power value that satisfies the scale-free topology characteristics and has reasonable connectivity was selected for constructing the co-expression network.

After quality control, this study used Weighted Gene Co-expression Network Analysis (WGCNA) to construct a gene co-expression network under salt stress time series based on the normalized expression data of 36 Xiaoyan22 samples. The dataset included 36 samples (6 time points × 2 treatments × 3 biological replicates), exceeding the commonly recommended minimum sample size (≥15) for reliable WGCNA module detection, thereby supporting network robustness. To satisfy the scale-free topology characteristics common in biological networks, different soft-threshold powers were first evaluated. The results showed that as the power value increased, the scale-free fit index gradually increased and stabilized; considering both the scale-free fit and mean connectivity, the optimal soft-thresholding power was determined to be β = 20, which achieved a scale-free topology fit index (R^2^) > 0.85 while maintaining appropriate mean connectivity (Figure 2). The above results indicate that the co-expression network constructed in this study conforms to the scale-free characteristics in its topological structure, providing a reliable basis for subsequent module identification and functional analysis.

### 3.3. Co-Expression Module Identification and Module Merging

**Figure 3:**
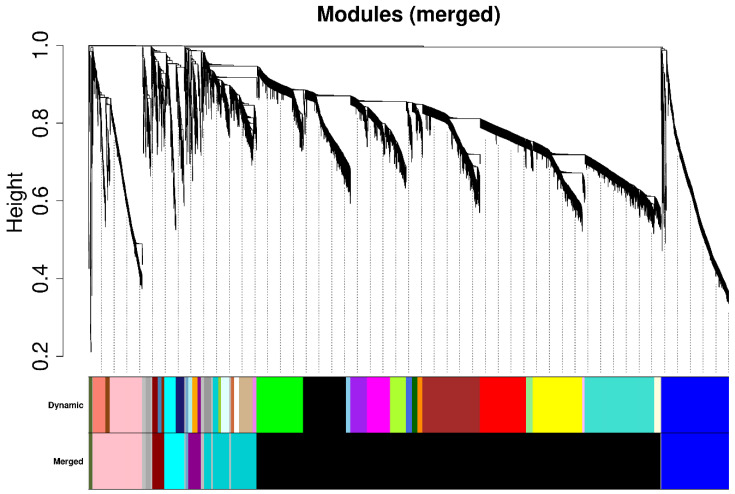
Gene clustering tree, module identification, and module merging results. Hierarchical clustering of genes was performed based on the topological overlap matrix (TOM), and co-expression modules were identified using the dynamic tree cutting algorithm. Different colors represent different modules; modules with high similarity were further merged to obtain the final module partitioning results.

Using a soft-threshold parameter derived from screening, this study subsequently generated a topological overlap matrix (TOM) and applied the dynamic tree-cutting algorithm to cluster genes into modules based on significant co-expression patterns. Clustering and module partitioning are shown through the gene dendrogram and module partitions. Each gene may be categorized into separate gene-sets defined by the color of the associated module according to the degree of co-expression; however, at the same time, modules with similar module eigengene (ME) were merged together in order to reduce redundancy between module clusters and create more stable clusters (Figure 3). A total of 11 modules were identified, one of which is the unassigned grey module. There were marked differences in the number of genes per module. The largest module, the black module, consists of 5050 genes, followed by the blue module consisting of 838, pink module consisting of 618, and darkturquoise module consisting of 609. In contrast, skyblue3 (32 genes) and darkolivegreen (43 genes) were smaller modules (Figure 3), consistent with the module size statistics. In summary, WGCNA successfully constructed a co-expression module framework under salt stress time series, laying the foundation for subsequent module–trait association analysis and key gene screening.

### 3.4. Correlation Analysis of Modules with Salt Stress Characteristics (Treatment/Time) Reveals Key Modules

**Figure 4:**
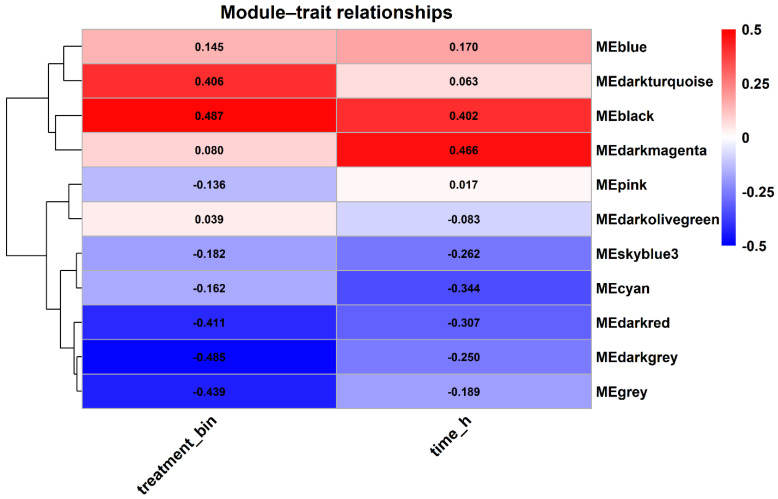
Heatmap of module–trait correlation analysis. This heatmap shows the correlation between module eigengenes (ME) and trait variables (salt treatment_bin and time_h). The color intensity indicates the direction and strength of the correlation. The correlation coefficient (r) and significance *p*-value are also shown, which are used to identify key modules closely related to the dynamic response to salt stress. The color scale was adjusted to emphasize correlation within the observed range (−0.5 to 0.5) for improved visualization.

To screen for key co-expression modules related to the dynamic response to salt stress, this study performed Pearson correlation analysis between sample trait information (treatment variable treatment_bin: CK = 0, Salt = 1; time variable time_h) and the module eigengenes (ME) of each module. The module–trait correlation heatmap showed that different modules had significantly different correlation directions and strengths with salt treatment and time progression (Figure 4). Among them, the black module showed a moderate to strong positive correlation with salt treatment (r = 0.487, adjusted *p* = 0.003) and with time progression (r = 0.402, adjusted *p* = 0.015), suggesting that this module may represent the core transcriptional response program of Xiaoyan22 that gradually strengthens over time during salt stress (Figure 4). In addition to the black module (r = 0.487, adjusted *p* = 0.402, adjusted *p* = 0.015 for time), the darkturquoise module also showed moderate positive correlation with treatment/time (r = 0.406, adjusted *p* < 0.05), while the skyblue3 module showed a negative correlation trend with treatment and time (r < 0, adjusted *p* < 0.05) (Figure 4), indicating that both promoting and inhibiting regulatory processes may exist simultaneously under salt stress conditions. Based on the strength of module–trait correlation and module size, this study identified the black module as the key salt-responsive module for further analysis.

### 3.5. Dynamic Expression Trajectories of Key Module Characteristic Genes over Time

**Figure 5:**
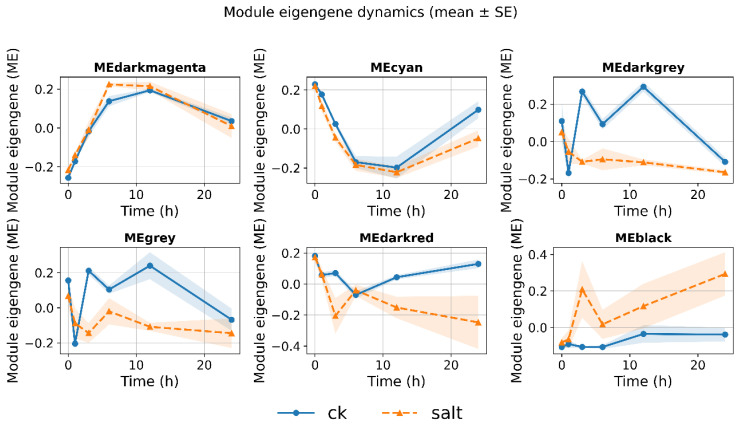
The dynamic trajectories of module eigengenes (MEs) across the salt stress time series are shown. This illustrates the expression trends of different module eigengenes (MEs) over time. Key modules exhibit continuously increasing or specific dynamic patterns under salt treatment conditions, revealing the potential functional stages of these modules during the stress adaptation process.

To characterize the dynamic response features of key co-expression modules during salt stress, this study further compared the changes in module eigengenes (ME) of each module at different treatment conditions and time points. The results showed that most modules exhibited varying degrees of dynamic fluctuations during the stress process; among them, the ME value of the black module continuously increased over time under salt treatment conditions, showing a gradually strengthening trend (Figure 5). This expression pattern is consistent with its positive correlation with both treatment_bin and time_h in the module–trait correlation analysis, further supporting the close relationship between the black module and the salt stress process (Figure 5). In summary, the black module may represent a core transcriptional response unit that is continuously activated in the later stages of stress in the salt-tolerant variety Xiaoyan22, suggesting its possible involvement in long-term homeostasis maintenance and adaptation processes, and is consistent with the phased dynamic characteristics usually observed in salt stress responses.

### 3.6. Black Module Hub Gene Screening and Identification of Key Genes Within the Module

**Figure 6:**
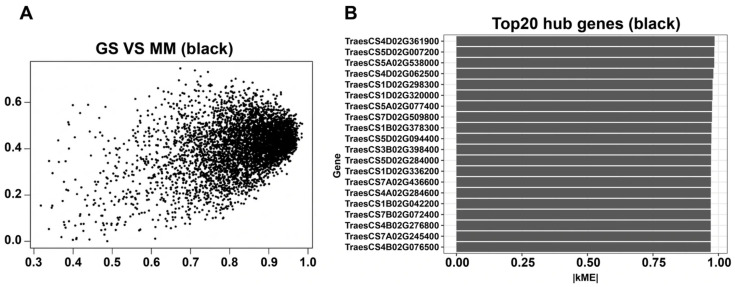
Hub gene screening and identification of key genes within the Black module. (**A**) Relationship between gene significance (GS) and module membership (MM) in the Black module. A scatter plot is shown illustrating the relationship between gene significance (GS, correlation with the trait) and module membership (MM, correlation with the module’s characteristic genes), using the black module as an example. (**B**) Expression patterns of the top 20 hub genes in the Black module. This shows the expression patterns of the top 20 hub genes, selected based on module membership (kME/MM), across the salt stress time series.

To identify potential hub candidate genes in the black module, this study calculated the module membership (MM; i.e., the correlation between gene expression and module eigengene) and gene significance (GS; i.e., the correlation between gene expression and phenotypic traits) for each gene within the module. The GS–MM scatter plot showed a significant positive correlation between GS and MM in the black module (r = 0.585, *p* = 3.03 × 10^−15^), indicating that genes with a higher correlation to salt stress traits often also have higher module connectivity, possessing the characteristics of key hub genes (Figure 6A). Based on this, this study ranked the genes according to kME (correlation with the module eigengene) to screen for candidate hub genes in the black module, and further displayed the expression patterns of the Top 20 hub genes (Figure 6B). The aforementioned hub genes exhibited relatively consistent dynamic response trends under salt treatment conditions, suggesting their potential crucial regulatory roles in salt tolerance adaptation. This provides a basis for the prioritized screening of candidate genes in subsequent functional validation and molecular breeding (Figure 6B).

### 3.7. GO Functional Enrichment of the Black Module Reveals Its Involvement in Key Biological Processes

**Figure 7:**
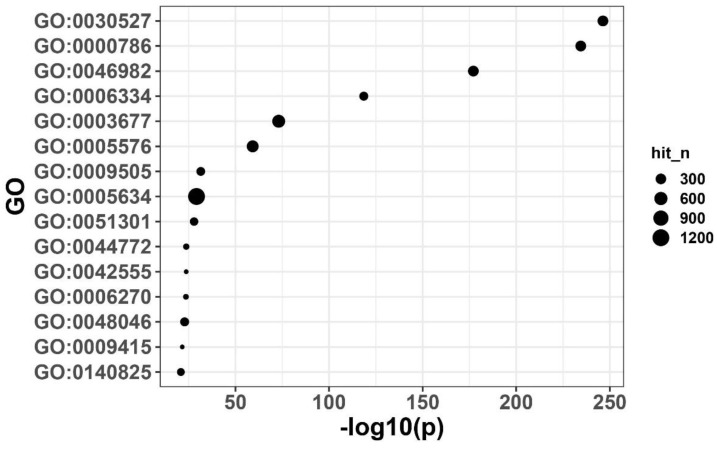
GO functional enrichment analysis of genes in the black module. Gene Ontology (GO) over-representation analysis (ORA) was performed on the genes in the black module, showing the top 15 significantly enriched GO terms. The x-axis represents enrichment significance or gene proportion, and the dot size represents the degree of enrichment or significance level, used to analyze the main biological processes and molecular functions of this module.

An over-representation analysis (ORA) of the Black module showed that it is enriched in every category of gene related to stress response and basic metabolic activity. Some examples of the functional categories where genes within this module were significantly enriched include the regulation of DNA replication initiation, the interaction of RNA with proteins, and the response to cold temperatures (cold acclimation). Each of these functional categories was statistically significant based on our multiple-testing correction (Figure 7). The enrichment of genes within the Black module suggests that not only do they contribute to the response to salt stress, but they are also likely involved in maintaining cellular homeostasis through the support of key biological processes such as DNA replication/repair, RNA metabolism and post-transcriptional regulation. This information provides molecular evidence supporting the continuing adaptation of salt-tolerant plants to salinity stress conditions (Figure 7).

### 3.8. Pathway Enrichment Analysis of the Black Module Reveals Its Core Metabolic and Regulatory Network Characteristics

**Figure 8:**
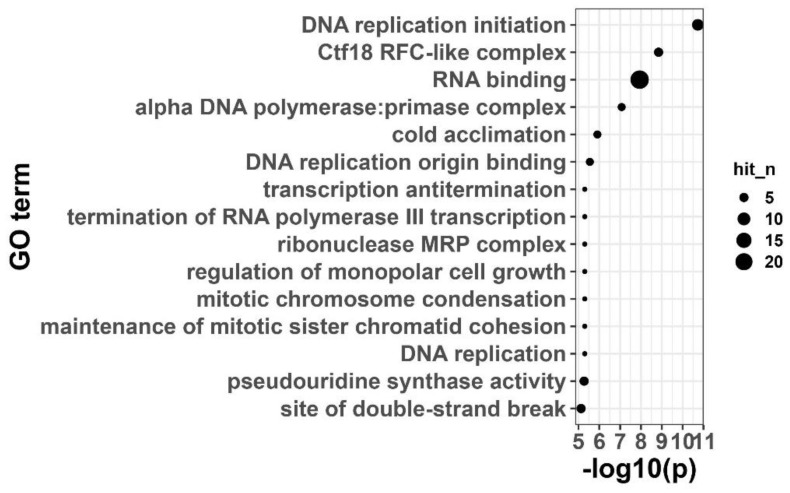
Pathway enrichment analysis of genes in the black module. Pathway enrichment analysis (KEGG/Pathway ORA) was performed on the genes in the black module, showing the top 15 significantly enriched pathways.

The black module underwent further pathway enrichment analysis (KEGG/Pathway ORA) and was found to be significantly enriched in multiple traditional pathways associated with stress adaptation (Figure 8). Pathways with the greatest enrichment in this module were DNA replication, phenylpropanoid biosynthesis, and cutin, suberin and wax biosynthetic pathways (Figure 8). Cell wall remodeling and antioxidant defense are heavily linked to phenylpropanoid metabolism [33]. On the other hand, cutin, suberin, and waxes are involved in the formation of barrier structures as well as minimizing water loss [34]. As a result, the black module enrichment suggests that through strengthening structural barriers and coordinating the regulation of metabolic pathways, they will most likely enhance the salt tolerance level in Xiaoyan22 (Figure 8). Furthermore, the black module was significantly enriched in pathways related to transcriptional regulation and protein synthesis, such as ATP-dependent chromatin remodeling and ribosome biogenesis (Figure 8), suggesting that salt tolerance responses may be accompanied by extensive chromatin remodeling and translational regulation processes to support transcriptional reprogramming and homeostasis maintenance under stress conditions.

### 3.9. Physiological Indicators and qRT-PCR Verification Under Salt Stress

**Figure 9:**
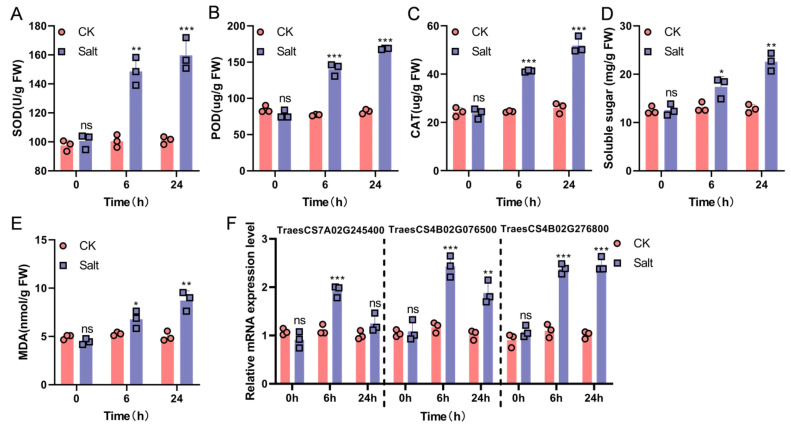
Physiological indicators and qRT-PCR validation under salt stress. Physiological responses and candidate gene expression validation of Xiaoyan22 under salt stress. (**A**–**E**) Salt treatment significantly increased the activity of antioxidant enzymes SOD (**A**), POD (**B**), and CAT (**C**), promoted the accumulation of soluble sugars (**D**), and led to an increase in MDA content (**E**) over time, but overall remained controllable; (**F**) Meanwhile, qRT-PCR showed that ZAR1-like receptor kinase, Remorin, and NETWORKED1D responded significantly under salt stress. Data are presented as mean ± SD (*n* = 3). ns represents *p* > 0.05, * *p* < 0.05, ** *p* < 0.01, *** *p* < 0.001.

Salt stress significantly induced the enhancement of the antioxidant defense system in Xiaoyan22. Compared with the control, SOD, POD, and CAT activities increased significantly after 6 h of salt treatment and further increased or maintained high levels at 24 h, indicating that Xiaoyan22 can quickly activate and continuously strengthen its ROS scavenging ability (Figure 9A–C). Meanwhile, osmoregulatory substances accumulated significantly under salt stress: proline content rapidly increased at 6 h and accumulated in large quantities at 24 h, and soluble sugar levels were significantly higher at both 6 and 24 h, indicating that it alleviates water imbalance and osmotic pressure caused by salt stress through continuous osmotic regulation (Figure 9D). In addition, MDA content gradually increased with stress time, suggesting that salt stress induced a certain degree of membrane lipid peroxidation; combined with the simultaneous enhancement of antioxidant enzyme activity, this indicates that Xiaoyan22 may limit further membrane damage to some extent by strengthening its antioxidant defense (Figure 9E).

At the molecular level, qRT-PCR verification showed that all three candidate genes exhibited a significant response to salt stress: ZAR1-like receptor kinase (TraesCS7A02G245400) was significantly upregulated at 6 h and then decreased at 24 h but remained higher than the baseline; remorin 4.1 (TraesCS4B02G076500) was upregulated at 6 h and maintained a high level at 24 h; NETWORKED 1D (TraesCS4B02G276800) was continuously and significantly upregulated from 6 to 24 h (Figure 9F), supporting its potential functions related to membrane signal transduction and structural homeostasis regulation. In summary, this study constructed a WGCNA co-expression network based on the time-series transcriptome of Xiaoyan22 under salt stress, and identified a key module, the black module, significantly associated with salt treatment and time course (Figure 4 and Figure 5). Highly connected hub genes were then selected from this module (Figure 6). Functional enrichment analysis indicated that this module is mainly involved in DNA replication and homeostasis maintenance, RNA metabolic regulation, as well as phenylpropanoid metabolism and cuticle/suberin and wax synthesis (Figure 7 and Figure 8). Combined with physiological indicators and candidate gene expression validation (Figure 9), the results collectively demonstrate that Xiaoyan22 exhibits enhanced antioxidant defense, accumulation of osmoregulatory substances, and significant responses of key genes under salt stress, providing an experimental basis for further analysis of salt tolerance regulatory mechanisms and the selection of candidate genes for molecular breeding.

## 4. Discussion

Salt stress limits wheat growth and yield through osmotic stress, ion toxicity, and excessive accumulation of reactive oxygen species (ROS) [8,35]. Numerous transcriptomic studies in wheat, including DEG-based analyses of salt-treated seedlings, have reported induction of ion transporters, antioxidant enzymes, and hormone-responsive genes as major components of the stress response [36,37,38]. However, these studies primarily emphasized differentially expressed genes at discrete time points and did not explicitly examine coordinated temporal regulatory programs.

Based on time-series transcriptome data of the salt-tolerant wheat variety Xiaoyan22 under salt stress, this study used WGCNA to construct a time-series-based co-expression network integrating temporal information. Unlike conventional DEG-based pipelines, which treat genes as independent units, the WGCNA groups genes into modules based on shared expression trajectories, thereby enabling the identification of coordinate transcriptional programs. Through this approach, a key module (the black module) was identified (Figure 4 and Figure 5), which showed significant positive correlation with both salt treatment treatment_bin and stress duration (time_h). High-connectivity hub genes were further screened within this module (Figure 6), combining functional enrichment with physiological and qRT-PCR validation. This study processes a system-level regulatory framework for salt tolerance in Xiaoyuan22 and provides candidate gene resources for future functional validation and molecular breeding. It is important to note that this study focused solely on the salt-tolerant genotype Xiaoyan22. Therefore, the identified black module may reflect a general salt stress response rather than being specific to tolerance mechanisms. Comparative analyses with salt-sensitive genotypes would be required to determine which components are truly tolerance-associated.

In contrast to earlier DEG-centered wheat studies that reported large sets of salt-responsive genes at h hg or 24 h without resolving their coordinated structure [36,37], the network-based reanalysis here reveals progressively activated gene modules associated with stress progression. This temporal modular perspective suggests that salt adaptation may involve sustained activation of interconnected transcriptional programs rather than isolated transcriptional bursts. From a dynamic perspective, the black module exhibited a continuous increase in eigengene expression under salt treatment (Figure 5), suggesting that it represents a sustained adaptive transcriptional program rather than a transient early response. Previous physiological and transcriptomic analyses have described salt stress responses as a phased process involving early osmotic sensing, signal transduction, and subsequent metabolic and structural adjustment [36,37,38]. However, not all classical stress-responsive genes were strongly associated with the black module, highlighting exceptions and the limitations of co-expression analysis in capturing the full spectrum of salt-induced responses. The progressively enhanced trajectory observed here is consistent with those staged models but further refines them by identifying a specific co-expression module that statistically integrates late-stage transcriptional coordination. This supports the hypothesis that sustained module activation may be more closely associated with tolerance establishment than short-term transcriptional spikes.

Functional enrichment analysis revealed that, in addition to classical stress-related pathways such as phenylpropanoid biosynthesis and cutin/suberin and wax biosynthesis (Figure 8), the black module was significantly enriched in DNA replication, chromatin remodeling, RNA binding, and ribosome biogenesis processes (Figure 7 and Figure 8). Previous wheat salt-stress transcriptome studies have predominantly emphasized ion transport (e.g., HKT transporters), ROS scavenging enzymes (SOD, CAT, POD), and ABA-related signaling components [36,39], whereas replication and chromatin-associated processes were not typically highlighted as central themes. Given the large size of the black module (~5050 genes), these broad enrichments may reflect generalized stress activation. Focusing on hub genes and sub-module cores can provide a clearer mechanistic picture of salt response, particularly signaling and regulatory nodes. The enrichment of genome stability and chromatin-related categories in the present network suggests that prolonged salt exposure may require maintenance of replication fidelity and sustained transcriptional capacity, thereby supporting long-term adaptive reprogramming rather than solely activating classical defense pathways.

Physiological validation further supported this integrative model. Salt stress significantly increased SOD, POD, and CAT activities and promoted proline and soluble sugar accumulation, while MDA levels, although elevated, remained controlled (Figure 9A–E). These results align with prior physiological reports in salt-tolerant wheat cultivars showing enhanced antioxidant defense and osmotic adjustment capacity under saline conditions [34,40]. Although the enriched pathways in the black module suggest potential contributions to these physiological traits, the direct gene-to-phenotype relationships remain speculative. Therefore, the proposed “network physiology integration” should be interpreted cautiously. The enrichment of phenylpropanoid metabolism is mechanistically consistent with enhanced antioxidant capacity and cell wall reinforcement, as phenolic compounds contribute to ROS scavenging and structural stabilization. Similarly, enrichment of cuticle and wax biosynthesis pathways supports barrier strengthening to reduce transpirational water loss and ion penetration. Together, these findings extend previous DEG-based observations by integrating structural reinforcement, metabolic regulation, and cellular homeostasis into a coordinated module-level framework.

Among high-MM hub genes, greater emphasis was placed on membrane-associated signaling components and regulatory proteins that may function upstream in stress perception. Gene significance (GS) and module membership (MM) were positively correlated within the black module (Figure 6A), indicating that genes most strongly associated with salt treatment were also topologically central. qRT-PCR validated three annotated hub candidates: a ZAR1-like receptor kinase (TraesCS7A02G245400), which was rapidly induced at 6 h, and remorin 4.1 (TraesCS4B02G076500) and NETWORKED 1D (TraesCS4B02G276800), which showed sustained upregulation from 6–24 h (Figure 9F). Receptor-like kinases have previously been implicated in abiotic stress perception and integration with Ca^2+^ and MAPK signaling cascades in wheat and other cereals [39]. The sustained induction of membrane microdomain-associated proteins such as remorin suggests potential remodeling of membrane signaling platforms during prolonged salt exposure. It should be emphasized that these hub genes are statistically central within the network and stress-responsive, but their functional roles in salt tolerance have not been confirmed. Future studies using genetic manipulation are required to validate causality. These observations refine existing salt signaling models by proposing a regulatory chain linking membrane signal perception, microdomain reorganization, and downstream homeostasis maintenance.

Several methodological limitations should be acknowledged. WGCNA assumes approximate scale-free topology and uses correlation-based associations, which may not capture nonlinear or condition-specific regulatory interactions. The 0–24 h sampling window captures early responses but may not represent long-term adaptation. Module detection is sensitive to parameter selection and network construction choices, which could affect hub gene identification. Overall, compared with previous DEG-centered wheat salt studies that focused primarily on ion transport, ROS detoxification, and hormone signaling pathways, the present work establishes a systematic association of “module–trait–hub gene–functional pathway” through WGCNA, integrates physiological validation, and highlights the importance of coordinated transcriptional modules and cellular homeostasis processes in salt tolerance adaptation. However, given the single-genotype design, the broad functional enrichment, and the correlative nature of hub gene identification, conclusions about salt tolerance mechanisms should be interpreted cautiously. This study provides a foundation for targeted functional analyses and comparative studies with additional.

## 5. Conclusions

**Figure 10:**
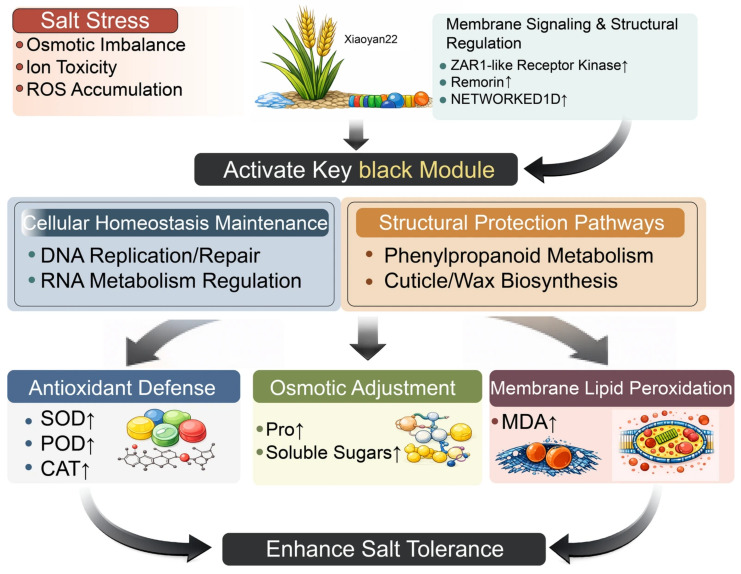
Hypothetical model of the salt stress-associated regulatory framework in the salt tolerant wheat cultivar Xiaoyan22. Salt stress triggers osmotic imbalance, ion toxicity, and ROS accumulation, activating key black modules through membrane signaling and structural regulatory factors (ZAR1-like receptor kinase, Remorin, NETWORKED1D). This module synergistically regulates cellular homeostasis maintenance (DNA replication/repair, RNA metabolism regulation) and structural protection pathways (phenylpropanoid metabolism, cuticle/suberin and wax synthesis), thereby enhancing antioxidant defense (SOD/POD/CAT) and osmotic regulation (Pro, soluble sugars), and promoting overall salt tolerance improvement while keeping MDA levels under control.

This study constructed a WGCNA co-expression network based on time-series transcriptome data of the salt-tolerant wheat variety Xiaoyan22 under salt stress. A key black module that was significantly associated with salt treatment and time progression, and whose activity increased with prolonged stress, was identified. The mechanistic diagram (Figure 10) shows that after salt stress triggers osmotic imbalance, ion toxicity, and ROS accumulation, membrane signaling and structural regulatory factors (such as ZAR1-like receptor kinase, remorin, and NETWORKED 1D) may mediate the activation of the key module, thereby synergistically regulating cellular homeostasis maintenance (DNA replication/repair, RNA metabolism regulation) and structural protection pathways (phenylpropanoid metabolism, cuticle/wax synthesis). Consistent with this, physiological results showed increased antioxidant enzyme activity, enhanced proline and soluble sugar accumulation, and elevated but overall controllable levels of membrane lipid peroxidation; qRT-PCR also validated the salt stress response of related candidate genes (Figure 9). In summary, this study proposes a hypothetical salt stress-associated regulatory framework of “membrane signal perception—key module activation—synergistic homeostasis and structural protection” and provides priority candidate gene resources for molecular breeding of salt-tolerant wheat.

## Data Availability

The RNA-seq dataset analyzed in this study was obtained from [24] and is publicly available in the China National Center for Bioinformation (CNCB) database under accession number PRJCA033247.

## Supplementary Materials

The following supporting information can be downloaded at: https://www.mdpi.com/article/10.3390/cimb48030317/s1.
